# Whole Wheat Bread Enriched with Cricket Powder as an Alternative Protein

**DOI:** 10.3390/foods11142142

**Published:** 2022-07-19

**Authors:** Ampaka Mafu, Sunantha Ketnawa, Suphat Phongthai, Regine Schönlechner, Saroat Rawdkuen

**Affiliations:** 1 Food Science and Technology Program, School of Agro-Industry, Mae Fah Luang University, Chiang Rai 57100, Thailand; 6131401029@lamduan.mfu.ac.th (A.M.); sunantha.ketnawa@gmail.com (S.K.); 2 School of Agro-Industry, Faculty of Agro-Industry, Chiang Mai University, Chiang Mai 50100, Thailand; suphat.phongthai@cmu.ac.th; 3 Cluster of High Value Product from Thai Rice for Health, Chiang Mai University, Chiang Mai 50100, Thailand; 4 Department of Food Sciences and Technology, Institute of Food Technology, BOKU-University of Natural Resources and Life Sciences, Muthgasse 18, A-1190 Vienna, Austria; regine.schoenlechner@boku.ac.at; 5 Unit of Innovative Food Packaging and Biomaterials, School of Agro-Industry, Mae Fah Luang University, Chiang Rai 57100, Thailand

**Keywords:** *Acheta domesticus*, alternative protein, cricket, edible insect, future food

## Abstract

The current market trends in modern sedentary lifestyles drive the development of new functional products able to fulfill consumers’ demand for a healthy diet. Whole wheat bread contains more protein and fiber than white bread; however, it could be improved in terms of protein content and quality. Cricket powder, which contains high protein (55.11, wt%), could be used as an alternative source to tackle those deficiencies in such bread. Hence, the study aimed to apply cricket powder in the whole wheat bread formula to enrich protein content, indispensable amino acids and determine their physico-chemical properties, consumers’ acceptance, and shelf-life storage. The results showed that all enriched bread presented high protein (18.97–25.94, wt%), fat (10.91–15.07, wt%), and ash (2.09–2.33, wt%) with the increment of the cricket powder than those of the control bread. Enriched breads’ crust colors were not significantly different (*p* > 0.05), while crumb colors were darker (L* = 55.64–64.48) compared to the control (L* = 69.98). Enriched bread had a hard texture and required a lot of chewing force compared to the control. Furthermore, all samples yielded a shelf-life of 5 days when monitoring the mold growth. From the results, the bread enriched with 20% cricket powder yielded the best consumers’ acceptance score of 77%. It was predominantly high in indispensable amino acids such as leucine, phenylalanine, lysine, and arginine. Therefore, cricket powder could be a novel alternative protein source and successfully utilized in whole wheat bread to enhance its protein content and indispensable amino acids with consumers’ acceptance responding to the current market trend.

## 1. Introduction

The steadily increasing global population will reach 9.8 billion people in 2050 [1] resulting in a high requirement for high-quality foods. However, the common protein source from livestock requires high resources, emits high greenhouse gasses, and threatens pandemics [2]. Therefore, it is imperative to look for alternative protein sources, such as insect protein, which demonstrates good protein quality, is economical, sustainable, and eco-friendly [3]. Nowadays, this could encourage the use of edible insects as a novel alternative protein source in the future because it contains not only high-quality proteins but fatty acid compositions, dietary fiber, vitamins as well as minerals [4,5,6]. Moreover, insect farming requires fewer resources such as land, water, and management compared with livestock farming, and both types of farming produce a similar amount of protein [7].

Bread is a vital staple food and is widely accepted and consumed throughout the world. Increasing bread consumption was reported as a 1.43% Compound Annual Growth Rate (CAGR) between 2019 and 2024 in the world market due to its convenience, portability, nutrition, and taste [8]. The statistic shows 192.68 million Americans consumed whole wheat bread in 2020, which takes first place followed by white bread (135.48 million) [9]. Among other kinds of bread, whole wheat bread provides a relatively high protein content of 11.48% [10]; however, it may lack certain indispensable amino acids as well as an inadequate amount of protein for consumers. This limitation could be tackled by enriching it with ingredients that contain high amounts of those nutrients. Mitelut et al. [11] documented the new trends relating to bread consumption, production, and consumer requirement which is the new functional bakery products able to satisfy new consumers’ demand for healthy bread, using different functional ingredients. Moreover, in terms of consumers’ perception regarding insect-based food, the consumers prefer to consume a product where the insects are not visible with the product providing high nutritional features and good palatability.

The research and development on the application of edible insects is being actively pursued and can be observed by a large number of patents and research articles. Currently, 167 patents and 488 articles related to the applications of edible insects have been filed [10]. In previous studies, wheat bread fortification with various edible insect flours, i.e., mealworm, buffalo worm, cricket [12,13], and the larvae of the black soldier fly [13] improved the biological value of bread due to the high protein content characteristics. Pauter et al. [14] applied 10% cricket powder to increase the protein content of muffins by 1.4-fold. In addition, da Rosa Machado and Thys [7] reported that the enrichment of cricket powder on gluten-free bread can lead to high protein content (8.53–12.52, wt%). The same trend was reported according to Nissen et al. [15], where cricket powder was reported to provide gluten-free sourdough bread with high nutritional value proteins. In addition to nutritional value, cricket enrichment helps products have a unique aroma [15] and improved texture, namely increased hardness and improved consistency [16].

There are some species of insects that are very popular to consume in Thailand such as house crickets, bamboo caterpillars, and grasshoppers [17,18]. Consequently, a house cricket (*Acheta domesticus*) was selected due to it being a good source of protein (55–70, wt%) [6], its affordable price, and all-year-round availability [17]. Additionally, the cricket has unique nutritional values such as indispensable amino acids, unsaturated fatty acids, vitamins, and minerals [5,6]. Moreover, it contains chitin and chitosan which can inhibit pathogenic microorganisms [19]. These advantages have proved that crickets are potentially suitable as a novel alternative protein source for humankind in the future. The work focused on the protein enrichment of whole wheat bread with cricket powder which fully hopes to respond to the recent trend of consumers’ demand for healthy bread. Additionally, the feasibility of the utilization of cricket powder in foods to fulfill the demand for high nutritive diets in a rapidly escalating world population and with increasingly limited natural resources is a challenging issue. Therefore, this study aimed to apply cricket powder to enrich the protein content of whole wheat bread and determine its physicochemical properties. Moreover, the sensory evaluation and shelf-life storage of the protein-enriched whole wheat bread were also evaluated.

## 2. Materials and Methods

### 2.1. Raw Materials

Frozen house cricket (*Acheta domesticus*) was purchased from the cricket farm (Bird Farm, Ban Du, Chiang Rai, Thailand). Pandan leaves were purchased from the local market of Ban Du, Chiang Rai, Thailand. The ingredients for the production of whole wheat bread were purchased from a baking supply shop (Anajak Bakery Supply, Muang, Chiang Rai, Thailand) including the main ingredients; whole wheat flour (whole wheat flour, 73% + wheat flour, 24%, Red Bell^®^, Imperial, KCG Corporation, Bangkok, Thailand), wheat flour (wheat flour 100%, Ma dang^®^, King milling, Samut Prakan, Thailand, and other ingredients; milk, unsalted butter, dried yeast, salt, sugar, and vanilla flavor.

### 2.2. Production of Cricket Powder

The frozen house cricket was placed in an aluminum tray and thawed in the refrigerator (10 °C) for 12 h before the production of cricket powder. Cricket powder was prepared using the method according to Lucas-González et al. [20] with slight modification. In brief, the thawed cricket was rinsed passes under running tap water, drained, and air-dried until an invisible drop of water was observed. Then, the cricket was separately placed in an aluminum tray. Fresh pandan leaves were added 15% by the weight based on the weight of fresh cricket to lend a unique aroma. The cricket was dried using a tray dryer (Model BP-80, KluayNamThai TwoOp, Bangkok, Thailand) at 60 °C for 13 h to obtain the moisture content of 1–5% by the weight. The dried cricket was ground using a hammer mill (Model CMC-20, Chiang Rai, Thailand) and sieved using a sieve tray size 1 mm (No. 18). Fine cricket powder was packed in a vacuum packaging and kept at -20 °C until analysis.

### 2.3. Determinations of Cricket Powder

#### 2.3.1. Determination of Physico-Chemical Properties

Determinations of chemical compositions including moisture, protein, fat, and ash content followed American Association of Cereal Chemists (AACC) approved methods of analysis [21]. Moisture content was performed by a thermogravimetric method in a hot air oven at 105 °C for 16 h (Method 44-15.0). Ash content was conducted in a muffle furnace at 525 °C (Method 08-01.01). Protein content was performed by the Kjeldahl method and the nitrogen converting factor is 6.25 (Method 46-13.01). Fat content was performed by a Soxhlet extractor (Method 30-25.01). Total carbohydrate content was calculated by subtracting the value of protein, ash, and lipid content from 100%.

Determination of physical properties including color using a slightly modified method according to Bawa et al. [22]. The cricket powder was measured by a colorimeter (Hunter Associates Laboratory, Reston, VA, USA). Results were shown as L*, a*, and b* scores. The L* indicated the lightness of the sample from blackness (L* = 0) to whiteness (L* = 100). The a* indicated red (a* is positive) and green (a* is negative) of the sample. The b* indicated yellow (b* is positive) and blue (b* is negative) of the sample.

#### 2.3.2. Determination of Functional Properties

Water holding capacity (WHC) and oil holding capacity (OHC) were determined by using a slightly modified method according to da Rosa Machado and Thys [7]. One gram of cricket powder was mixed with 10 mL of water or soybean oil to determine a WHC and OHC, respectively. The centrifuge tube was allowed to stand at room temperature for 30 min and centrifuged at 3500 rpm for 20 min. The WHC and OHC were reported as the amount of water/oil absorbed per g of sample. The WHC and OHC were calculated as follows:

$$\begin{array}{c} \mathrm{WHC}\;\left(g/g\;\mathrm{sample}\right)\mathrm{or}\;\mathrm{OHC}\;\left(g/g\;\mathrm{sample}\right)\\ = ((\mathrm{Weight}\;\mathrm{of}\;\mathrm{the}\;\mathrm{tube}\;\mathrm{with}\;\mathrm{supernatant}\\ \;-\mathrm{Weight}\;\mathrm{of}\;\mathrm{the}\;\mathrm{tube}\;\mathrm{without}\;\mathrm{supernatant}))/\left(\mathrm{Weight}\;\mathrm{of}\;\mathrm{sample}\;\mathrm{used}\right) \end{array}$$

Foaming capacity (FC) and foaming stability (FS) were conducted by a slightly modified method by Stone, Tanaka and Nickerson [4]. Two grams of sample were dispersed with 50 mL water and adjusted pH to 7. Samples were shaken overnight using a shaker. The mixture sample was readjusted pH to 7, left to stand for 1 h, and homogenized at 10,000 rpm for 1 min. The samples were poured into a graduated cylinder and measured the volume at time zero (FC) and 30 min (FS).

$$\mathrm{FC}\;\left(\%\right)=\lfloor \frac{\left(\mathrm{Volume}\;\mathrm{after}\;\mathrm{homogenization}-\mathrm{Volume}\;\mathrm{before}\;\mathrm{homogenization}\right)}{\mathrm{Volume}\;\mathrm{before}\;\mathrm{homogenization}}\rfloor \times 100$$

For foam stability, the samples were allowed to stand for 30 min at room temperature and the remaining foam volume was measured. The following formula was used to calculate FS:

$$\mathrm{FS}\;\left(\%\right)=\left[\left(\mathrm{Residual}\;\mathrm{foam}\;\mathrm{volume}\;\mathrm{at}\;30\;\min\right)/\left(\mathrm{Total}\;\mathrm{foam}\;\mathrm{volume}\;\mathrm{at}\;\mathrm{time}\;0\;\min\right)\right]\times 100$$

### 2.4. Production of Whole Wheat Bread Enriched with Cricket Powder

Whole wheat bread was prepared following a formula presented in Table 1 according to Solangi et al. [23] and da Rosa Machado and Thys [7] with slight modification. All dried ingredients (whole wheat flour, wheat flour, cricket powder, sugar, yeast, and salt) were poured into a mixer bowl and mixed using a whisk. The mixture was transferred to dough kneading machine (Model 5KPM5EWH, KITCHENAID^®^, St. Joseph, MI, USA) and mixed at speed No. 4 for 15 min. while vanilla flavor and milk were gradually added until the dough was obtained. Then, the mixer bowl was covered with plastic wrap, and the dough left to ferment at 40 °C for 1 h. After dough fermentation, the dough was removed by a rolling pin and molded-in aluminum bread mold tray (6.7 cm width × 13.7 cm length × 5.5 cm height) then covered with plastic film wrap and let stand for 20min. The dough was baked at 175 °C for 30 min then the loaves removed from the bread mold tray. Loaves were left to cool down at ambient temperature for 2 h. All loaves were packed in polyethylene bags and stored at −40 °C until analysis.

### 2.5. Determinations of Whole Wheat Bread Enriched with Cricket Powder

#### 2.5.1. Determination of Physico-Chemical Properties

Determinations of chemical compositions including moisture, protein, fat, ash, and carbohydrate content were performed as previously described above.

Amino acid profile was performed only in the cricket powder enriched bread with the best acceptance score and determined using outside analysis service by Central Laboratory (Chiang Mai, Thailand) Co., Ltd. employing the in-house method TE-CH-372 based on the Official Journal of the European Journal of communities, L257116 by amino acid analyzer technique.

Physical properties in terms of the color of crust and crumb and texture profile including hardness, cohesiveness, springiness, and chewiness were determined. The color of crust and crumb were determined followed the method described by Bawa, Songsermpong, Kaewtapee and Chanput [22], Loave samples were sliced to 1 cm thickness and determined the color of crust and crumb using a colorimeter and evaluated the L*a*b* score; L* (0 is blackness, 100 is whiteness), a* (positive is red, negative is green), b* (positive is yellow, negative is blue). Texture profile analysis (TPA) was determined using a slightly modified method according to da Rosa Machado and Thys [7]. Loaves were sliced to 1 cm height and measured the TPA using a texture analyzer (TA.XT plus Texture Analyzer, UK) and analyzed data including hardness, cohesiveness, springiness, and chewiness. Calibration with 50 kg load cell and a P/75 probe. Test parameters included pre-test speed: 2 mm/s, test speed: 1.7 mm/s, post speed: 2 mm/s and compression test: 80%.

#### 2.5.2. Shelf-Life Evaluation

Determination of the shelf-life of loaves was conducted following the method described by Kamaljit [24]. Loaves samples were packed in polyethylene bags and stored at ambient temperature (28–30 °C). During storage, the loaves were observed for mold growth on the surface of slices and data recorded every day for 7 days.

#### 2.5.3. Sensory Evaluation

Sensory evaluation was conducted at the Food Sensory Lab, Department of Food Science and Technology, School of Agro-Industry, Mae Fah Luang University. Ethical approval for the consumer testing and evaluation of the products was granted by Mae Fah Luang University, Chiang Rai, Thailand. The consent of all participants was achieved. Participants for the consumer testing and evaluation of the bread samples comprised 30 untrained panelists, 15 females and 15 males. The panel was asked to rate their preference of how they like the appearance, texture, flavor, taste, and overall acceptability of the products by indicating a numerable value to rate their preference from one (expressing dislike extremely) to nine (like extremely). They were also asked to rate their acceptability of the products via nine points hedonic from one (dislike extremely) to nine (like extremely). To obtain a good understanding of consumer opinions, this stage was evaluated by 100 untrained panelists in the sample which provided the best score from the previous evaluation and commercial whole wheat bread. The panelists were also requested to provide comments and suggestions regarding product development.

### 2.6. Statistical Analysis

All data analyses were investigated triplication and expressed as mean ± standard deviation. The different formulas were analyzed using one-factor analysis of variance (ANOVA) by the Statistical Tool for Agricultural Research (STAR) software program (International rice research institute, Manila, The Philippines). The significance level of *p* < 0.05 was considered significantly different.

## 3. Results and Discussion

### 3.1. Characteristics of Cricket Powder

#### 3.1.1. Physico-Chemical Properties

The chemical composition of the cricket powder was presented on a dry weight basis (g/100 g, d.b.) including moisture 2.77 ± 0.11, protein 55.11 ± 3.10, lipid 22.83 ± 0.40, ash 4.80 ± 0.10, carbohydrate 17.26 ± 0.23, respectively. Cricket powder predominantly consists of protein and lipid. The same results were reported by Rumpold and Schlüter [6], Mariod, Saeed Mirghani and Hussein [5] and Lucas-González, Fernández-López, Pérez-Álvarez and Viuda-Martos [20], who reported 55.00–70.75%, 9.80–22.80%, 3.57–9.10% and 16.35–22.08% for protein, lipid, ash and carbohydrate, respectively. Although cricket powder contains high lipids, the lipids are considered indispensable or functional fatty acids which provide health benefits. According to Mariod, Saeed Mirghani and Hussein [5], house cricket is reported to contain essential fatty acids such as linoleic (30–40%), oleic (23–27%), palmitic (24–30%), and stearic acid (7–11%).

The color of cricket powder showed L* (40.81 ± 0.61), a* (4.9 ± 0.53), b* (15.13 ± 2.02), respectively. The resulting value indicates that the cricket powder has a dark color due to the luminosity (L*) which indicates the lightness is quite low. The same result was reported by other researchers including Lucas-González, Fernández-López, Pérez-Álvarez and Viuda-Martos [20] who stated that thermally dried cricket powder showed a low value in L* (41.62 ± 0.61). In another study, Bawa, Songsermpong, Kaewtapee and Chanput [22] reported that the obtained cricket powder from the oven baking showed L* (54.93 ± 0.2). The color could be different depending on cricket species and cooking process.

#### 3.1.2. Functional Properties

The applicability of such protein ingredients depends upon their potential to fulfill one or more functional requirements, viz. solubility, water holding capacity (WHC), and oil holding capacity (OHC) or foam and emulsion stabilization [25]. WHC and OHC are one of the most critical functional properties of proteins attributed to the physical entrapment of water or oil and influence the texture quality and interaction between the water and oil of the product [25]. Liquid retention is an index of the ability of proteins to absorb and retain oil/water which in turn influences the texture and mouth feel characteristics of the products [26]. This study found that cricket powder showed a WHC of 7.39 ± 0.09 g/g sample and OHC of 7.34 ± 0.17 g/g sample which are higher than that of the study according to da Rosa Machado and Thys [6], who reported 2.87 ± 0.04 g/g and 3.22 ± 0.26 g/g, respectively. Cricket powder would therefore be added to the bakery products and helps maintain technological properties.

The foaming capacity (FC) and foaming stability (FS) of cricket powder were 8.1 ± 1.20% and 32.43 ± 9.89%, respectively. It was reported that foamability is related to the rate of decrease in the surface tension of the air/water interface caused by the absorption of protein molecules. Low foamability could be related to highly ordered globular proteins, which resist surface denaturation [25]. Ndiritu et al. [27] stated that house cricket has poor FC (11.11%) and FS (10.05%). Additionally, Akpossan et al. [24] presented that no FC and FS of the full-fat flour of caterpillar larvae according to high lipid content which may have also prevented foaming [4,28]. Foams are used to improve the texture, consistency, and appearance of foods [25]. In this study, cricket powder may not be suitable for the application of the product that required those properties. Improved foamability of cricket powder, whether in defatted powder or isolated protein powder, may need to be further investigated.

### 3.2. Characteristics of Whole Wheat Bread Enriched with Cricket Powder

#### 3.2.1. Physico-Chemical Properties

The results of moisture, ash, protein, lipids and total carbohydrate on a weight dry basis (g/100 g) are depicted in Table 2. In particular, moisture, lipid, and ash content were significantly increased in all the samples where cricket powder had been added. The moisture content of bread enriched with cricket powder for 10% and 25% was the highest and significantly different (*p* < 0.05) from the control bread. The moisture content seemed inconsistent with the linear increase in cricket powder. During kneading, protein absorbs water to enable it to flex into gluten. The more protein in the flour, the more water is required to effectively hydrate the dough [29]. However, the dough mixing between gluten protein as well as cricket protein molecules is gradually hydrated but inconsistent as without cricket powder. This may be the reason for the fluctuation of moisture content in enriched bread which needs to be investigated in the future. For the ash content, all loaves showed a higher content when increasing the amount of cricket powder due to the ash content of cricket powder (4.67 ± 0.10) which caused the enriched bread to have a high ash content. Moreover, the enriched loaves were reported to escalate in protein and lipid content by 1.2–1.65 times compared to the control due to the increment amount of cricket powder. Thus, cricket powder supplementation contributed to an increase in protein and lipid content of the enriched bread. The loaves containing cricket powder presented a lower carbohydrate value than the control. The cricket addition at 30% of the formula presented the highest protein content (25.94% by weight) but the lowest carbohydrate content (56.65% by weight) due to the mass balance. In previous studies, wheat bread fortification with various edible insect flours improved the protein content of bread in different ranges depending not only originates from differences between species and developmental stages but also on different feed and origins as well as differences in measuring methods [6]. Cricket powder-enriched bread could be a good choice of alternative protein-enriched bread for consumers because of its high protein and minerals. Cricket flour contains high protein and is low in carbohydrates, which is beneficial for obesity or diabetes patients. High protein food can increase insulin without an increase in blood sugar levels [30].

#### 3.2.2. Amino Acid Compositions

Amino acid compositions were determined only in the bread enriched with 20% cricket powder which yielded the highest acceptance score from panelists and is depicted in Table 3. Amino acids are the substituents of proteins that can indicate the quality of the protein source. Different protein sources contain different types of amino acids and influence the quality of protein [31]. This formula contains predominantly indispensable amino acids in leucine (1166.30 mg/100 g). Whereas wheat flour contains leucine of only 0.953 g/100 g [32], thus the escalation of leucine could come from the addition of cricket powder in the formulation. Duan et al. [33] stated that high-quality protein foods contain leucine because it is the most abundant amino acid in animal nutrition. The total indispensable amino acids of the enriched bread with 20% cricket flour were 2.06 mg/g protein, while the dispensable amino acids were 3.05 mg/g protein. When compared to the data from the literature on whole wheat bread [26], the enriched whole wheat bread with 20% cricket powder from the study predominantly showed the similar type in all amino acids (Table 3) because of the occurrence of indispensable amino acids in the cricket powder [6].

#### 3.2.3. Physical Properties

Texture profile analysis (TPA) showed that all cricket powder enriched loaves have a higher value of hardness (33,695.79–59,285.75 g) and chewiness (14,411.83–23,708.64 g) than control (Table 2). This can indicate that enriched loaves presented a hard texture and require a lot of chewing force compared with the control. However, the 10% cricket formula is not significantly different from the control. Bread loaves should have a high value of cohesiveness because this parameter refers to the ability to hold the materials [29]. The enrichment of cricket powder has a rich fat content (22.83% by weight) resulting in the crumble of the enriched bread. Moreover, a high amount of fat can affect the structure and physical properties, i.e., texture. This leads to a low gluten network and less retained gas bubbles which influence texture properties [30]. Springiness refers to the ability to return to the original shape of food. A slight decrease in springiness was reported in the enriched bread. Similar results on chewiness, cohesiveness, and springiness but different hardness values were reported by Pauter, Różańska, Wiza, Dworczak, Grobelna, Sarbak and Kowalczewski [14] who studied the replacement of wheat flour with cricket powder in muffins. According to Kowalczewski, Walkowiak, Masewicz, Bartczak, Lewandowicz, Kubiak and Baranowska [16], cricket powder altered both the bound and bulk water fractions through analyses of water behavior at the molecular level via 1H Nuclear Magnetic Resonance (NMR). Cricket protein could delay hydration and the development of the gluten network, leading to an increase in hardness [34]. Furthermore, an effect on hardness is due to the higher presence of ash (cations) [26]. Moreover, the addition of protein components could act on various gluten proteins directly or through some reaction by promoting the formation of additional interprotein bonds [35]. Therefore, the ratio of ingredients of bread formulations should be optimized to obtain the desired texture of baked goods and the consumers’ acceptability.

**Figure 1:**
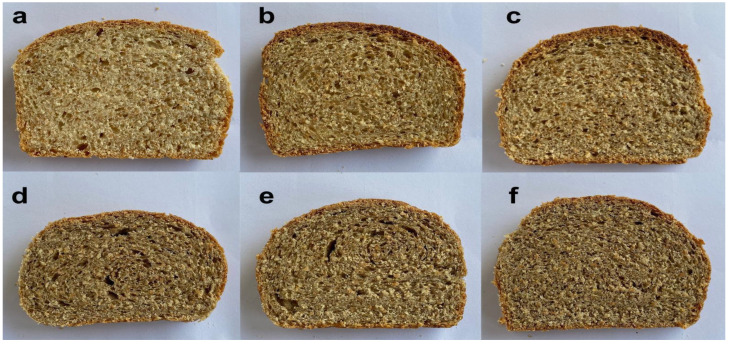
Images of the cross-section of bread slices: (**a**) control, (**b**) enriched bread with 10% cricket powder, (**c**) 15%, (**d**) 20%, (**e**) 25%, and (**f**) 30% by flour weight.

Images of the cross-section of bread slices are depicted in Figure 1. The obtained enriched bread has a darker color of crust and crumb than the control. All formulas also presented a darker crust. Moreover, the enriched bread formulas found that 10% cricket has the highest luminosity (L*) of both crust and crumb color. Cricket powder displayed a dark color (L* = 40.81 ± 0.61) which consequently meant enriched bread presented a dark color. Furthermore, the high protein content of cricket powder results in a high rate of Maillard reaction which is a chemical reaction of protein reacting with reducing sugar during heating. This reaction also enhanced the brown color and flavor [36].

### 3.3. Shelf-Life Evaluation

**Figure 2:**
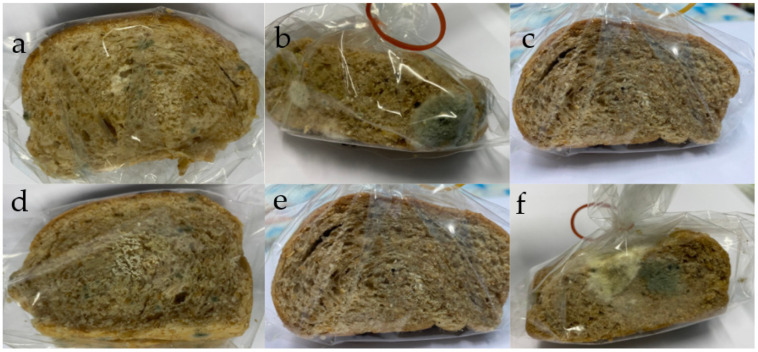
The mold growth on the cross-section of bread slices during shelf-life evaluation on the 5th day of storage at ambient temperature. (**a**) control, (**b**) enriched bread with 10% cricket powder, (**c**) 15%, (**d**) 20%, (**e**) 25%, and (**f**) 30% by flour weight.

Generally, homemade bread has a shelf-life of 4–6 days under ambient conditions [37]. In this study, all loaves had a shelf-life of 5 days after the first production date by the observation of mold growing on the surface of the bread slice (Figure 2). By the observation, bread loaves obtained on the production date exhibited a soft texture, good aroma, and dark color depending on the amount of cricket powder added. After shelf-life evaluation for 7 days, all bread slices presented a hard texture, bad smell, and a mold on the surface of the bread slices. Moreover, the oil inside the slice was released to the outside area. Smith [38] reported that the bread stored in plastic bags at 22 °C had a shelf-life of 5–6 days. Bread is a bakery product that classifies as a high moisture content product (aw = 0.96–0.98). The factors that influence the mold spoilage of bakery products are moisture content, temperature, humidity, packaging material as well as the surrounding gas environment [38].

The limitation of edible insects can be perceived by biological hazards associated with the rearing environment and insect’s gut. The presence of spore-forming bacteria in insect-based foods represents a serious threat to consumers’ health since these microorganisms are food-borne pathogens [39]. However, other spore-forming bacteria have not been investigated in the study. Osimani et al. [40] reported that spore-forming bacteria in cricket-based bread highlighted potential safety issues. To the author’s knowledge, Osimani, Milanović, Cardinali, Roncolini, Garofalo, Clementi, Pasquini, Mozzon, Foligni, Raffaelli, Zamporlini and Aquilanti [40] first characterized the spore-forming microorganisms in bread produced with insect powder from house crickets. In this study, baked bread loaves were subjected to viable counting of spore-forming bacteria and reported up to 3.7 log cfu/g, depending on the substitution level. Moreover, the presence of different Bacillus species was observed and documented including *Bacillus licheniformis*, *Bacillus subtili* and *Brevibacillus borstelensis*. The first two species are associated with ropy spoilage of bread and *B. licheniformis* is also potentially pathogenic to humans. Another point is that allergens are a major safety concern surrounding edible insects. Several research groups have identified potential antigens and IgE binding proteins in various insect species, which may correlate to an allergic reaction after consumption [41]. The guidelines from FAO publication [39] stated that the biological hazard risk could be mitigated by controlling cleanliness throughout the farming process. Eating the edible insect that passed a heat process and not consuming wild edible insects can also reduce this risk. For the ready-to-eat products, they can be clean and importable if they are prepared by the standard method and a mature industrial line.

### 3.4. Sensory Evaluation

The result showed that consumers favor the 20% cricket formula the most. Although the results of the 10%, 15%, and 20% cricket formulas were similar in some characteristics such as appearance, texture, color, flavor, taste, and overall acceptability which are demonstrated in Table 4. Although the overall acceptability of enriched bread with 10% cricket flour was not significantly different (*p* < 0.05) from that of enriched bread with 20% cricket flour, the enriched bread with 20% cricket flour was selected for the further step because of the higher protein content. In the comparison among all bread, it was found that enriched bread with 20% cricket flour yielded a higher score on the overall acceptability. In terms of the food industry and marketing of the product, sensory evaluation with a high overall acceptability score could be used as the key point due to one of the most significant factors influencing the consumer purchase decision and consumption [42]. As reported by Burt, Kotao, Lopez, Koeppel, Goldstein, Samuel and Stopler [30], insect-based foods where insects are not directly visible and provide high nutritional features with good palatability could be more acceptable by consumers. Moreover, one of the most challenging issues for the industrial sector is related to making consumers respond with acceptance and positive attitudes toward innovative insect-based foods.

Commercial whole wheat bread was reported to contain total energy (260 Calories), total fat (4%), protein (2%), total carbohydrate (44%), and dietary fiber (4%). It was found that the acceptance score of 77% was derived from the appearance, texture, color, flavor, taste, and overall acceptability of enriched bread with 20% cricket flour compared to those of commercial bread which is 100%. Nevertheless, consumers suggested improving the quality of the product in terms of texture since cricket powder contains high lipid content, thus, making the bread quite crumbly. Balancing the ratio of ingredients and reducing fat content should be improved in future studies.

## 4. Conclusions

Cricket powder can be utilized as a novel alternative protein source in terms of protein quality, and as an environmentally friendly resource. All enriched bread formulas presented high protein content and the one enriched with 20% cricket powder had the best acceptance without any effect on shelf-life. The derived products successfully combine the compositional and sensory characteristics with some peculiar nutritional properties of cricket. Cricket powder can constitute a novel source of innovative ingredients to be used for the protein enrichment of indispensable amino acids and minerals. In future studies, fatty acid profile analysis is advisable to confirm the functional fatty acids contained in the starting material and final product. Future investigation should also consider improving the texture as well as insect bioactive properties within the food matrix and the effect on allergenicity. Lastly, the current work is not only in response to the current trends in modern sedentary lifestyle with the development of the new functional products able to fulfill consumers’ demand for a healthy diet, but the economics of the country as well. The edible cricket industry will be expanded by adding up the value of processed products. Moreover, bakery industries and entrepreneurs also yield the outcome from this study with the invention of functional health products. The world is also impacted in terms of world food security and environmental issues because insect farming is more pre-eminent in terms of lower greenhouse gas emissions, water, and soil use, but with higher feed conversion and edible mass compared to domestic livestock. Moreover, making consumers respond with positive attitudes toward innovative insect-based foods is one of the most challenging issues facing the industrial sector.

## Figures and Tables

**Table 1:** Formulation of whole wheat bread enriched with cricket powder.

Ingredients (g)	Control (0)	Cricket Powder (%, *w/w* Flour Basis)				
		10	15	20	25	30
Main ingredients						
Whole wheat flour	40	40	40	40	40	40
Wheat flour	60	60	60	60	60	60
Cricket powder	0	10	15	20	25	30
Other ingredients						
Milk	75	75	75	75	75	75
Unsalted butter	15	15	15	15	15	15
Sugar	8	8	8	8	8	8
Yeast	4	4	4	4	4	4
Salt	0.6	0.6	0.6	0.6	0.6	0.6
Vanilla flavor	1	1	1	1	1	1
Total ingredients	203.6	213.6	218.6	223.6	228.6	233.6

**Table 2:** Nutritional compositions and physical properties of control and cricket powder-enriched whole wheat bread.

Sample	Control	Cricket Powder (%, *w/w* Flour Basis)				
	0	10	15	20	25	30
Nutritional properties (based on a dry basis)						
Moisture (g/100 g)	27.12 ± 0.02 ^bc^	29.16 ± 0.13 ^ab^	23.33 ± 0.10 ^d^	26.60 ± 0.04 ^c^	31.24 ± 0.03 ^a^	28.46 ± 0.01 ^bc^
Protein (g/100 g)	15.72 ± 3.57 ^d^	18.97 ± 0.37 ^c^	21.90 ± 0.46 ^b^	22.79 ± 0.07 ^b^	25.86 ± 0.95 ^a^	25.94 ± 0.10 ^a^
Lipids (g/100 g)	9.07 ± 0.07 ^c^	10.91 ± 0.09 ^bc^	10.33 ± 1.30 ^bc^	11.85 ± 0.18 ^abc^	13.89 ± 0.22 ^ab^	15.07 ± 1.15 ^a^
Ash (g/100 g)	1.67 ± 0.02 ^c^	2.09 ± 0.13 ^b^	2.22 ± 0.10 ^a^	2.34 ± 0.04 ^a^	2.21 ± 0.03 ^a^	2.33 ± 0.01 ^a^
Carbohydrate (g/100 g)	73.53 ± 0.07 ^a^	68.03 ± 0.76 ^b^	65.55 ± 0.72 ^b^	63.01 ± 1.79 ^c^	58.04 ± 0.34 ^d^	56.65 ± 1.39 ^d^
Color						
L* crust	52.47 ± 4.69 ^ab^	56.18 ± 0.63 ^a^	53.80 ± 1.62 ^ab^	55.90 ± 0.60 ^a^	51.57 ± 1.20 ^ab^	49.91 ± 1.22 ^b^
a* crust	12.66 ± 0.61 ^a^	10.10 ± 0.51 ^b^	10.57 ± 0.77 ^b^	9.23 ± 0.28 ^b^	9.57 ± 0.32 ^b^	9.22 ± 0.23 ^b^
b* crust	21.50 ± 0.44 ^a^	12.38 ± 2.23 ^d^	18.63 ± 0.69 ^ab^	17.05 ± 0.52 ^bc^	15.39 ± 0.82 ^cd^	13.24 ± 0.63 ^d^
L* crumb	69.98 ± 1.63 ^a^	64.48 ± 0.98 ^b^	61.30 ± 0.42 ^c^	58.18 ± 1.67 ^d^	57.96 ± 0.44 ^d^	55.60 ± 0.60 ^d^
a* crumb	3.70 ± 0.24 ^c^	4.78 ± 0.17 ^c^	5.22 ± 0.15 ^ab^	4.39 ± 0.06 ^a^	4.68 ± 0.22 ^b^	4.68 ± 0.14 ^b^
b* crumb	18.03 ± 0.77 ^a^	17.48 ± 0.24 ^a^	17.22 ± 0.27 ^a^	14.37 ± 0.29 ^b^	13.59 ± 0.97 ^b^	13.84 ± 0.36 ^b^
Texture properties						
Hardness (g)	31,840.97 ± 123.42 ^f^	33,695.79 ± 69.04 ^d^	33,363.07 ± 121.79 ^e^	44,886.30 ± 62.31 ^c^	55,170.31 ± 41.94 ^b^	59,285.75 ± 95.64 ^a^
Cohesiveness	0.77 ± 0.03 ^ab^	0.80 ± 0.02 ^a^	0.73 ± 0.00 ^b^	0.62 ± 0.01 ^c^	0.61 ± 0.01 ^c^	0.63 ± 0.02^c^
Springiness	0.77 ± 0.03 ^a^	0.54 ± 0.01 ^c^	0.58 ± 0.02 ^c^	0.62 ± 0.01 ^b^	0.61 ± 0.01 ^b^	0.61 ± 0.02 ^b^
Chewiness (g)	12,422.32 ± 92.48 ^f^	14,411.83 ± 81.12 ^e^	16,820.48 ± 71.84 ^d^	17,765.58 ± 79.47 ^c^	23,708.64 ± 174.07 ^a^	18,313.16 ± 58.98 ^b^

Results were expressed as the average of the triplicate samples with mean ± SD. Within each line, the mean values superscripted by different letters in the same row are significantly different (*p* < 0.05).

**Table 3:** Amino acid compositions (mg/100 g dry basis) of the enriched whole wheat bread with 20% cricket powder (the highest acceptability).

Amino Acid (mg/100 g)	Cricket 20% Formula (mg/100 g)	mg/g Protein	Amino Acid (mg/100 g)	Cricket 20% Formula (mg/100 g)	mg/g Protein
	Indispensable Amino Acids			Dispensable Amino Acids	
Threonine	530.87	23.29	Aspartic acid	1241.24	54.46
Valine	829.67	36.41	Serine	807.25	35.42
Methionine	<200.00	<8.78	Glutamic acid	3755.95	164.80
Leucine	1166.30	51.18	Alanine	858.30	37.66
Phenylalanine	975.18	42.79	Proline	1350.97	59.28
Histidine	455.72	19.99	Cysteine	247.31	10.85
Lysine	734.46	32.23	Tyrosine	493.91	21.67
Arginine	751.43	32.97			
Isoleucine	584.34	25.64			
Tryptophan	177.30	7.78			

**Table 4:** The sensory evaluation from 30 and then 100 panelists consumed the control and whole wheat bread enriched with cricket powder.

Sample	Control (0)	Cricket Powder (%, *w/w* Flour Basis)					Bread Sample	
		10	15	20	25	30	Cricket 20%	Commercial
Appearance	7.47 ± 1.22 ^a^	6.67 ± 1.09 ^ab^	6.37 ± 1.16 ^bc^	6.37 ± 1.40 ^bc^	5.20 ± 1.52 ^d^	5.59 ± 1.45 ^cd^	6.85 ± 1.61 ^b^	7.89 ± 1.27 ^a^
Texture	6.60 ± 1.57 ^a^	6.53 ± 1.45 ^a^	5.53 ± 1.41 ^ab^	6.13 ± 1.16 ^a^	4.60 ± 2.37 ^b^	4.83 ± 1.63 ^b^	6.33 ± 1.86 ^b^	7.86 ± 1.09 ^a^
Color	7.60 ± 1.19 ^a^	6.70 ± 1.32 ^ab^	5.87 ± 1.20 ^bc^	6.30 ± 1.58 ^b^	4.90 ± 1.67 ^cd^	4.76 ± 1.28 ^b^	6.68 ± 1.54 ^b^	7.81 ± 1.33 ^a^
Flavor	6.80 ± 1.35 ^a^	6.20 ± 1.69 ^ab^	5.40 ± 1.61 ^b^	6.37 ± 1.50 ^ab^	5.67 ± 1.97 ^ab^	5.45 ± 1.96 ^b^	6.64 ± 1.76 ^b^	7.42 ± 1.44 ^a^
Taste	7.10 ± 1.30 ^a^	6.03 ± 1.54 ^ab^	4.87 ± 1.25 ^bc^	5.93 ± 1.64 ^ab^	5.03 ± 2.04 ^bc^	4.45 ± 1.83 ^c^	6.26 ± 1.82 ^b^	7.69 ± 1.35 ^a^
Overall	7.47 ± 1.07 ^a^	6.60 ± 1.30 ^ab^	5.70 ± 1.44 ^bcd^	6.43 ± 1.52 ^abc^	5.47 ± 2.01 ^cd^	5.21 ± 2.85 ^d^	6.52 ± 1.47 ^b^	7.81 ± 1.09 ^a^
Acceptance (%)							77	100

Result were expressed as average from all tests reported as mean ± SD. Within each line, mean values superscripted by different letters in the same row are significantly different (*p* < 0.05).

## Data Availability

Not applicable.
